# Evaluation of alternative school feeding models on nutrition, education, agriculture and other social outcomes in Ghana: rationale, randomised design and baseline data

**DOI:** 10.1186/s13063-015-1116-0

**Published:** 2016-01-20

**Authors:** Aulo Gelli, Edoardo Masset, Gloria Folson, Anthoni Kusi, Daniel K. Arhinful, Felix Asante, Irene Ayi, Kwabena M. Bosompem, Kristie Watkins, Lutuf Abdul-Rahman, Rosanna Agble, Getrude Ananse-Baden, Daniel Mumuni, Elisabetta Aurino, Meena Fernandes, Lesley Drake

**Affiliations:** International Food Policy Research Institute (IFPRI), 2033 K Street NW, Washington, DC 20006 USA; Institute of Development Studies, University of Sussex, Brighton, BN1 9RE UK; Noguchi Memorial Institute for Medical Research (NMIMR), College of Health Sciences, University of Ghana, Legon, Greater Accra Region Ghana; Institute of Statistical, Social and Economic Research (ISSER), University of Ghana, Legon, Greater Accra Region Ghana; Partnership for Child Development (PCD), Department of Infectious Disease Epidemiology, Imperial College, London, W2 1PG UK

**Keywords:** School feeding, Impact evaluation, Education, Nutrition, Agriculture

## Abstract

**Background:**

‘Home-grown’ school feeding programmes are complex interventions with the potential to link the increased demand for school feeding goods and services to community-based stakeholders, including smallholder farmers and women’s groups. There is limited rigorous evidence, however, that this is the case in practice. This evaluation will examine explicitly, and from a holistic perspective, the simultaneous impact of a national school meals programme on micronutrient status, alongside outcomes in nutrition, education and agriculture domains.

The 3-year study involves a cluster-randomised control trial designed around the scale-up of the national school feeding programme, including 116 primary schools in 58 districts in Ghana. The randomly assigned interventions are: 1) a school feeding programme group, including schools and communities where the standard government programme is implemented; 2) ‘home-grown’ school feeding, including schools and communities where the standard programme is implemented alongside an innovative pilot project aimed at enhancing nutrition and agriculture; and 3) a control group, including schools and households from communities where the intervention will be delayed by at least 3 years, preferably without informing schools and households. Primary outcomes include child health and nutritional status, school participation and learning, and smallholder farmer income. Intermediate outcomes along the agriculture and nutrition pathways will also be measured. The evaluation will follow a mixed-method approach, including child-, household-, school- and community-level surveys as well as focus group discussions with project stakeholders. The baseline survey was completed in August 2013 and the endline survey is planned for November 2015.

**Results:**

The tests of balance show significant differences in the means of a number of outcome and control variables across the intervention groups. Important differences across groups include marketed surplus, livestock income, per capita food consumption and intake, school attendance, and anthropometric status in the 2–5 and 5–15 years age groups. In addition, approximately 19 % of children in the target age group received some form of free school meals at baseline.

**Conclusion:**

Designing and implementing the evaluation of complex interventions is in itself a complex undertaking, involving a multi-disciplinary research team working in close collaboration with programme- and policy-level stakeholders. Managing the complexity from an analytical and operational perspective is an important challenge. The analysis of the baseline data indicates that the random allocation process did not achieve statistically comparable treatment groups. Differences in outcomes and control variables across groups will be controlled for when estimating treatment effects.

**Trial registration number:**

ISRCTN66918874 (registered on 5 March 2015).

## Background

School feeding programmes have been a key response to the recent food and economic crises and function to some degree in nearly every country in the world [1]. School feeding is a multi-sectoral intervention with effects across education, health and nutrition, and with the potential for benefits across a life course. Rigorous studies have shown that school feeding programmes can improve school attendance and learning, as well as a child’s physical and psycho-social health (see [2] for a recent review). These effects are heterogeneous and context-specific, depending also on the quality of programme implementation. There is no rigorous evidence on the impact of providing a reliable market for smallholder farmers through ‘home-grown’ school feeding (HGSF) approaches [1, 2]. In HGSF, the demand for food and services from school feeding is channelled explicitly to smallholder farmers and other stakeholders involved in the school feeding supply chain. As most of the studies in the scientific literature in low-income settings involve humanitarian aid, there is also a paucity of evidence on government-led programmes operating at scale in low- and middle-income countries [1]. This study is aimed at addressing these research gaps by evaluating the full cost and impacts of alternative school feeding implementation approaches, across education, health and nutrition, and agriculture domains in Ghana.

### Country context

Ghana is a lower-middle income country with a population of 25 million people, over 40 % of whom are under 15 years of age [3]. Despite the high rates of economic growth occurred in the past two decades, Ghana is ranked 138th in the 2014 Human Development Index table, with a life expectancy at birth of 61 years, 7 mean years of schooling for adults and a Gross National Income (GDP) based on per capita purchasing power parity (PPP) of US$3532 [4]. The domestic economy is centred on subsistence farming, which accounts for nearly 40 % of the GDP and employs over 50 % of the workforce [5]. Around 25 % of the country’s population live in poverty based on the national-level poverty line, with this percentage increasing to 38 % in rural areas in contrast to 10 % in urban ones [6]. Food security in the marginal agricultural and arid areas varies with the seasons. The peak hunger seasons for the south of Ghana are from May to August whereas the north of Ghana experiences peak hunger seasons between July and October. The incidence of malnutrition in Ghana has been assessed through the Ghana Demographic and Health Surveys (GDHS) conducted every 5 years since 1988. From 1993 to 2008 there was some progress in reducing the rate of chronic malnutrition, with rates of stunting decreasing from 34 % to 29 % [6]. According to the 2003 and 2008 GDHS the prevalence of anaemia among children of 6–59 months of age increased marginally from 76 % in 2003 to 78 % in 2008. In 2008, the prevalence of anaemia among rural children aged under 5 years (84 %) was higher than in urban areas (68 %). The overall prevalence of stunting among school-aged children was 17 %, ranging from 13 % in the forest-savannah transitional zone to 21 % in the northern savannah [6]. The same study estimated that the prevalence of anaemia among school-aged children was 39 %. This, however, varied widely across ecological zones. Anaemia rates were highest in the northern savannah (65 %) and the coastal savannah zones (59 %) and least prevalent in the transitional zone (16 %).

### Complex intervention

This evaluation focusses on the Government of Ghana school feeding programme. As of 2011, the Ghana School Feeding Programme (GSFP) reached over 1.6 million primary school children in all 170 districts of Ghana. The programme is directly funded by the Government of Ghana, with a 4-year programme budget of over US$200 million. The GSFP was piloted in 10 schools in late 2005. By the end of 2009, GSFP had progressively grown to serve 1695 public schools with 656,624 pupils across the country. The GSFP is a complex intervention and was designed as a strategy to increase domestic food production, household incomes and food security in deprived communities [7]. The objectives of the strategy combined child-level education and nutrition, alongside household food production. GSFP co-ordination and implementation are undertaken by a national secretariat, with programme oversight provided by the Ministry of Local Government and Rural Development (MoLGRD). Line ministries offer technical support through the programme steering committee, although a number of NGOs and bilateral agencies are also involved with that support. The GSFP service delivery is provided through private caterers who are awarded contracts by the GSFP to procure, prepare and serve food to pupils in the targeted schools. Each caterer is responsible for procuring food items from the market, preparing school meals and distributing food to pupils. Cash transfers are made from the district assemblies, under the supervision of the District Implementation Committees (DICs), to caterers based on 40 Ghana pesewas (circa US$0.33) per child per day. Caterers are not permitted to serve more than three schools each, and profit is derived from savings made after food has been procured, prepared and distributed. Supervision at the school level is by the School Implementation Committee (SIC) and funds are intended to be released to caterers every 2 weeks. Storage is the responsibility of caterers and no rigid tendering process is enforced. The caterers are not restricted or guided in their procurement and are able to procure on a competitive basis without commitment to purchasing from small-scale farmers. The GSFP project document prioritises procurement from the community surrounding the assisted schools, broadening the focus to the district and national levels when food items are not available.

A recent supply chain analysis describes how caterer procurement decisions depend on costs (of food, transport, preparation) and on cash availability [8]. According to this study, the way and the extent to which caterers store food varies from district to district, but most have access to storage facilities (small household storage, school storage, or private storage). Caterers generally hire cooks to prepare food for students either in their homes or at school facilities. The main challenges faced by caterers include managing changes in food prices, hampered by the inability to mitigate price fluctuations due to delays in payments from the GSFP. Caterers reported that seasonal price variations between harvest and lean periods included price increases of up to 400 % [8]. The GSFP payments are received after the meals are served, resulting in caterers not having the resources to buy in bulk and guarantee a better and stable price to smallholder producers. Caterers were also reported to buy on credit from traders known as ‘market queens’ in Ghana, weakening their overall negotiation position. In addition, caterers also reported that payments often do not reflect the real number of pupils since enrolment often increases during the school term, which could possibly lead to either less food being served per child or higher costs faced by the caterers [9]. In practice, caterers often adapt to these challenges by reducing the quantity of food provided or by adjusting the quality of the food and adapting the menus. According to the supply chain study, procurement of food from smallholder farmers could help to mitigate the price volatility challenge. The study found that caterers were willing to procure their food from local farmers and that by buying from farmers, caterers could benefit from lower and more stable prices than those offered by traders on the market. Nonetheless, the reality is that almost all the food is still bought from markets [8].

### Challenges in linking agriculture

**Fig. 1:**
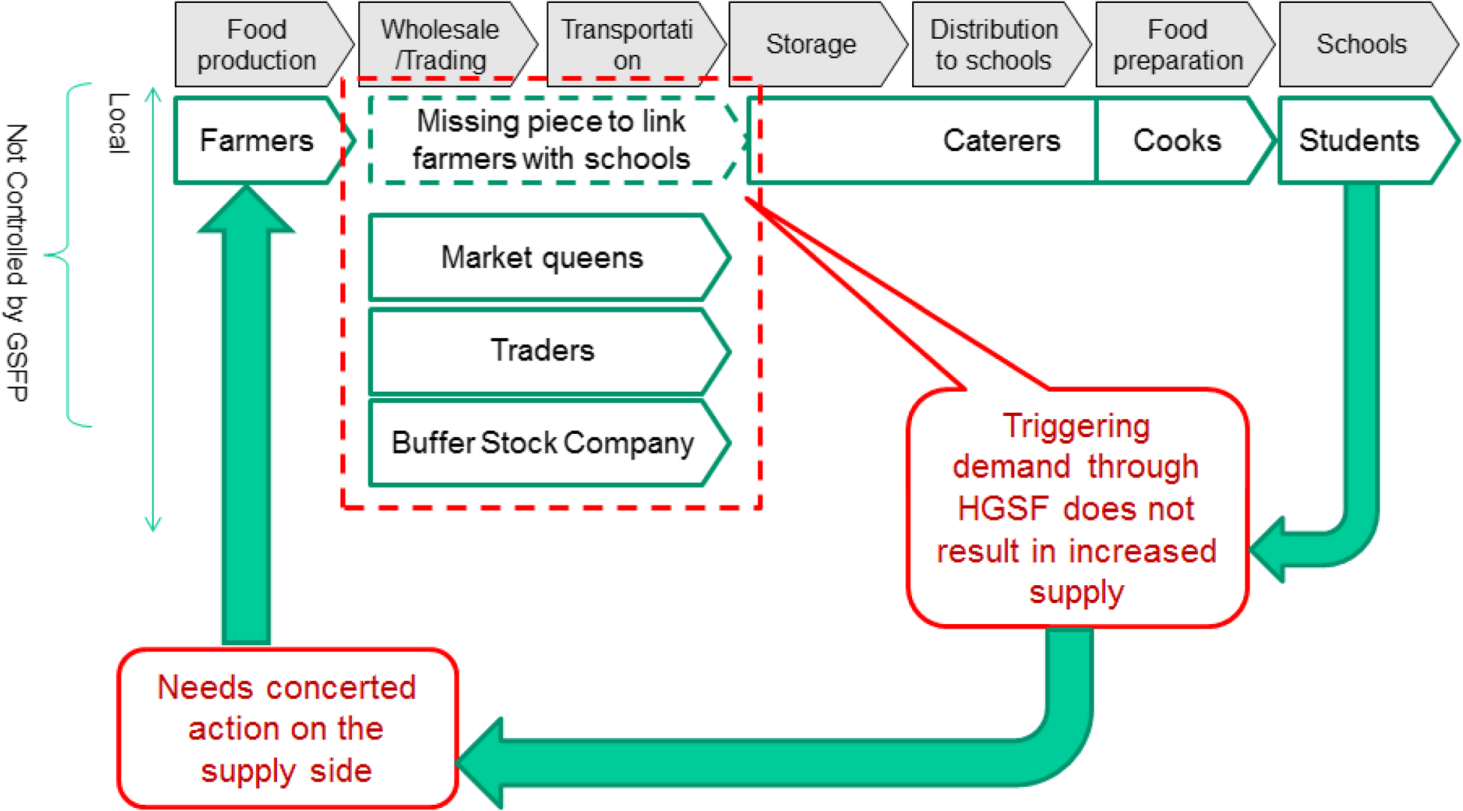
Missing link in the Ghana School Feeding Programme (GSFP) supply chain [8]

The most recent evaluation of the GSFP undertaken in 2012 identified the need for ‘a more strategic approach in linking farmers to the programme’ [10]. This gap between the food production side and the caterers has been documented in other studies as well, including a recent supply chain analysis that highlighted a number of key constraints in the current model (Fig. 1), including:

- Mismatch of cash flow: farmers need money as soon as they harvest. Caterers receive money after serving children
- Lack of trust between farmers and caterers (especially for future payments): farmers do not trust caterers to advance food for later payment. Inconsistent payment from government worsen their perceptions
- Difficult for caterers to access farmers: no contact information, difficult to reach, widely spread out, a lot of interaction necessary
- No structure in place to facilitate caterer and farmer negotiations

### The HGSF pilot

An innovative capacity-building component is being integrated alongside the GSFP and constitutes one of the treatment arms of the experiment. The details of the pilot were developed by a multi-disciplinary working group composed of in-country stakeholders under government leadership. This pilot involves the development of an integrated package of community-level activities aimed at enhancing the impact of the GSFP on poverty and food insecurity and involves two main components [11].

- Agriculture: this component is designed to stimulate the economy at community level by purchasing food from smallholder farmers. The component aims to bring the actors of the school feeding supply chain and GSFP community programme together to discuss the demand and supply needs to the school feeding market. Farmers and caterers would then be able to negotiate a price and payment agreement to address the issue of mistrust. This agreement will be backed by a master contract
- Nutrition: this component will include activities to improve the nutritional quality of the school meals (e.g. menu planning), promotion of improved health, nutrition and hygiene behaviours (e.g. behaviour change campaigns), and the provision of multiple micronutrient fortification

## Methods

### Programme theory of the intervention

School feeding interventions linked to smallholder agriculture can have multiple goals in the following areas:

- Education: increasing school enrolment, attendance and reducing drop-out, and improving cognition and learning achievement
- Health: improving nutritional status of school age children
- Agriculture: supporting incomes of recipient households (those consuming food) and farmer households (those providing the food)
- Small enterprise development: supporting incomes of caterers and cooks involved in the food service provision

**Fig. 2:**
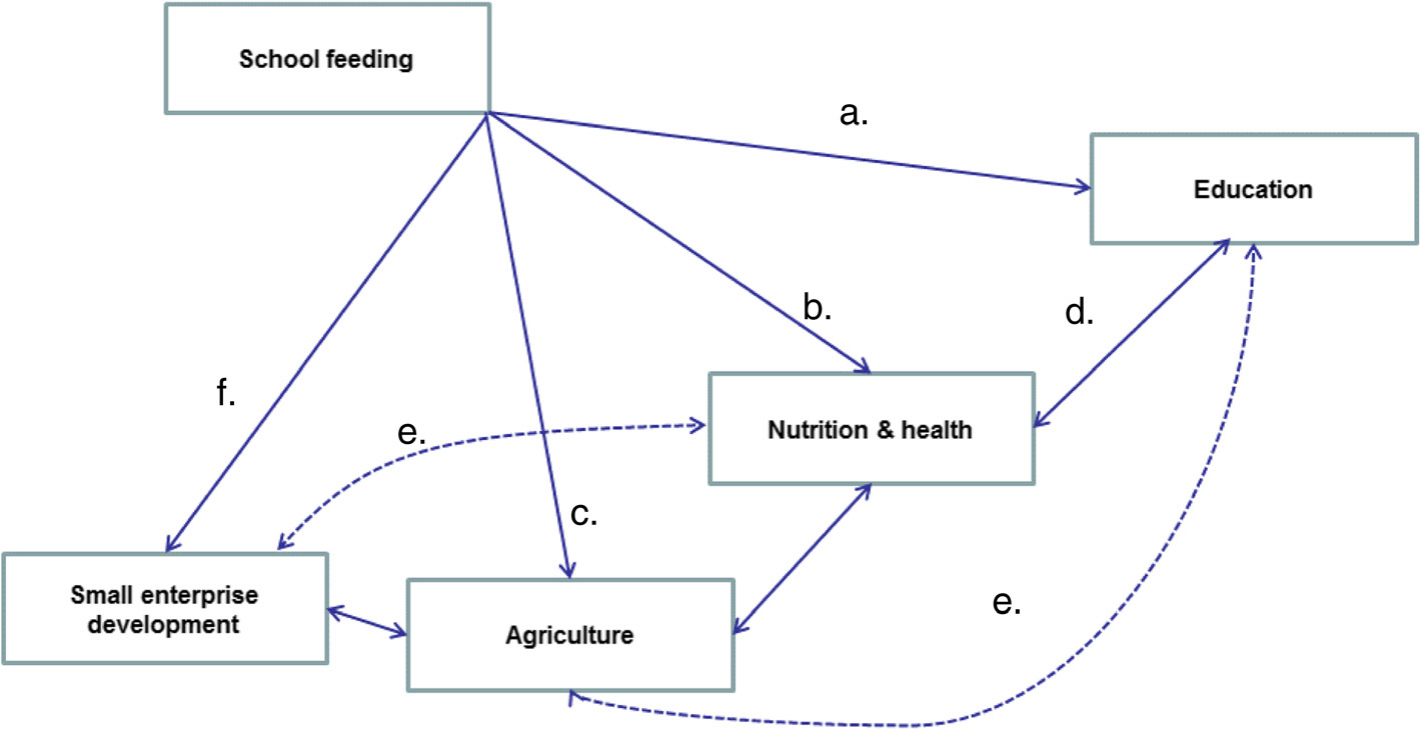
Overall programme theory of school feeding interventions

Figure 2 illustrates in very broad terms the impact theory of school feeding on agriculture, education, and health. School feeding affects educational outcomes directly by increasing enrolment, attendance and completion (line ‘a’ in the figure). It affects health directly by improving nutritional status (line ‘b’); this in turn has an indirect impact on education, as improving nutritional status has a positive impact on learning outcomes (line ‘d’). The intervention can also affect income directly by increasing households’ food security (line ‘c’). In addition, the intervention can benefit the small enterprises involved in the school food service provision. Finally, there are effects running through increased income and health and nutrition and vice versa, as richer families are investing more in human capital and more educated and healthier adults are more economically productive (lines ‘e’). However, these latter effects (represented as dotted lines in Fig. 2) only occur in the long term and certainly not before children have left school: therefore, we will not discuss them in the following design. Whilst the evidence base on the effects on child education, health and nutrition is generally well-established (see [12] for a recent systematic review) this evaluation is the first to also examine the effects on agriculture and enterprise development.

It must be emphasised that the ability of the school feeding intervention to deliver the effects depicted in Fig. 2 critically depends on the appropriate implementation of the programme. The management and implementation of the intervention involves several actors, and there is evidence that in Ghana there are several problems of information flow, supervision and monitoring between these different stakeholders. Programme success will also depend on the ability of communities to actively engage in the programme and in the strengthening of the public institutions involved.

### Main hypotheses and outcome indicators

We summarise here the expected impact of the intervention on education, nutrition and agriculture as captured in the programme theory. The detailed programme theory for the different domains is captured in [13].

### Education

- The intervention will have a positive impact on enrolment, attendance and drop-out rates
- The intervention will have an impact on cognitive abilities and class behaviour including attention
- The impact on learning (test scores) will be moderate as school quality is unlikely to change in the short term

### Nutrition and health

- The intervention will have a limited impact on physical growth of children because of the increase in physical activity levels (PAL), substitution effects and the age range (5–15 years) of the targeted population. An impact on siblings of school-going children is possible if substitution effects are strong
- The intervention will have a moderate impact on the diet because on the one hand, food purchases by caterers do not follow nutritional guidelines, and on the other nutrition education will be a component of the school-level trainings
- The intervention will have some impact on micronutrient status where the food provision is fortified, and only moderate effects on diet diversity are expected

### Agriculture and community development

- The intervention will have an impact on a small number of farmers in the intervention communities. Other persons in the community may benefit either directly or indirectly via an increase in income
- The programme will have an impact on a small number of caterers involved in the school feeding service provision

In addition to examining the potential effects in the different domains, the evaluation will also assess the pathways through which these effects are mediated.

Table 1 includes a list of the main outcome indicators of the study. The data collection section below describes how data will be collected using different survey instruments. All the main study outcomes, including school enrolment, attendance and test scores, will be obtained through the household- and child-level interviews.

**Table 1:** Primary indicators for the evaluation

Type	Domain	Indicator
Impact	Agriculture	Household income, production, sales
	Education	Child enrolment, attendance, completion, maths and literacy scores (5–15 year olds)
	Cognition	Raven’s test and forward/backward digit span scores (5–15 year olds)
	Physical health/Nutrition	Anthropometry (height-for-age, BMI-for-age, 2–15 year olds), haemoglobin levels (5–15 year olds)
Outcome	Food consumption	Nutrient adequacy and dietary diversity score (individual and household)
Output	Meal service	Quality of school meals, portion sizes, frequency and timeliness

For the pathways analysis, in addition to the outcome indicators in Table 1 we will also observe the programme impact on intermediate indicators, particularly for those outcomes that are more difficult to observe directly. In the case of farmer income, we will look at several intermediate outcomes such as input use (labour, land, seeds and fertiliser), investments (farm capital such as tools and machinery), and market access (marketed surplus, prices and markets). In terms of other intermediate indicators in the nutrition and health pathway, we will observe the effect of the programme on knowledge and practices of caterers and school management members, and on the quantity, quality, and timeliness of the preparation and delivery of the school meals.

### Design of the randomised evaluation

The impact evaluation will be an integral component of the monitoring and evaluation activities of the GFSP. Two rounds of surveys are envisioned, with the baseline planned in the intervention and control sites in June 2013 and a follow-up planned in November 2015. After the follow-up survey, the control schools and community will be fully integrated in the intervention. We will consider the possibility of conducting further surveys in the following years, building matched control groups in order to detect long-term effects of the intervention on smallholder agriculture.

The GSFP will be expanded across the 10 regions of the country. The GSFP has set clear criteria for the selection of the intervention areas as captured in the retargeting exercise conducted in 2012. Poverty rankings were developed using the Ghana Living Standards Survey and Core Welfare Indicators Questionnaire carried out in 2005/2006 and 2003 respectively. Food consumption scores were calculated using the Comprehensive Food Security and Vulnerability Assessment 2008/2009 and spatial data variables computed by the World Food Programme (WFP). The data were then used to generate district-level composites for share of national poverty and food insecurity that were then used to allocate programme resources.

### Random assignment and manipulation of treatments

Households and schools were randomly assigned to three treatment arms:

1. Control group: these are schools and households from communities where the intervention will not be implemented. The intervention will be delayed by at least 3 years in these communities, preferably without informing schools and households. After the 3- year period, these schools will be covered by the GSFP.
2. Regular GSFP group: these are schools and communities where the standard GSFP is implemented, with caterers responsible for the food procurement and preparation
3. HGSF+ group: these are schools and communities where the programme is implemented in addition to a pilot capacity-building component, including training of community-based organisations and other stakeholders, on food procurement, nutrition education, and feedback monitoring. This group will be randomly divided into two sub-groups (HGSF+ and HGSF++) as part of a study focussing on anaemia.

Note that the HGSF+ intervention will be conducted at the district level. Training and monitoring systems involve caterers and exert their effects at the district level, affecting outcomes in schools where the HGSF+ programme is not implemented. On the other hand, the number of districts where the programme is implemented is rather small, which reduces the statistical power of the analysis, and the effects of the school feeding intervention against the control group are best observed at the school level. Hence, we opted for a design that compares the outcomes of the school feeding and control groups at the school level, and that compares outcomes of HGSF+ and regular school feeding (GSFP) at the district level.

The GSFP selected 58 districts in which the programme will be implemented. In each of these districts, two candidate schools were selected and each school was randomly assigned to the treatment or to the control. A protocol was designed in order to ensure that the schools were comparable based on data from the Education Management Information system (EMIS) and that contamination between the two schools in each district will be minimised. This will allow comparison of outcomes of the intervention against the control group at the school level in 58 districts. The 58 schools assigned to the intervention were then randomly assigned to regular GSFP and HGSF+. In this way the randomisation of the HGSF+ intervention occurs at the district level. The number of 58 schools is based on power calculations (see Appendix 1) determined with the objective of achieving statistical validity and representativeness for the main outcomes of interest.

### Anaemia sub-study

**Fig. 3:**
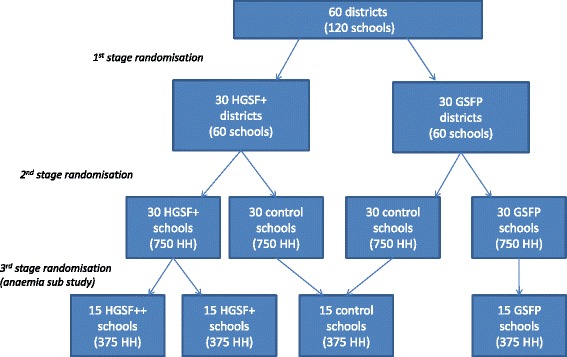
Schematic view of the design of the randomisation

The impact evaluation includes a sub-study focussing on nutrition in school feeding with and without micronutrient fortification. A sub-group of 14 of the 29 HGSF+ groups was randomly assigned to receive food fortification (the HGSF++ group) in addition to training and sensitisation activities that are part of the HGSF+ pilot (see Fig. 3). Data will be collected from children aged 5–15 years in the HGSF++, HGSF+, GSFP and control communities. Targeted schools were surveyed as part of the broader impact evaluation baseline.

### Sample sizes

For the impact evaluation, power calculations and resource availability suggested the adoption of a sample of 25 households from the communities in the areas of the 58 schools receiving the intervention and of 20 households in the communities of the 58 control schools.

Households were randomly selected from household listings in the catchment areas of the selected schools for the survey interviews. The household listings were stratified into farmer/non-farmer households, based on agriculture classification data from the national census. Farmer households were sampled in both areas in the following way: 10 out of the 25 households in the 60 intervention communities were farmer households and 5 out of the 20 households in the 60 control communities were farmer households. Non-farmer households with children in the 5–15 years age group were randomly selected from the household listings. This distribution of the sample between farmer and non-farmer households and between project control groups allows the construction of comparable samples (see Table 2).

**Table 2:** Sample sizes

	Districts	Schools	Households with children in the 5–15 years age group	Farmer HH	Children^a^
Control	58	58	870	290	2375
GSFP	29	29	435	290	1383
HGSF+	29	29	435	290	1383
(HGSF++)	(14)	(14)	(210)	(140)	(668)
Total	58	116	1740	900	5142

^a^the number of children is an estimate based on an average of 2.28 children per family in families with children and 1.35 children per family in farmer households

*GSFP* Ghana School Feeding Programme, *HGSF* ‘home-grown’ school feeding, *HH* households

In each household, all children aged between 5 and 15 years were asked education outcome-related questions (enrolment, attendance, drop-out) and were tested in literacy, maths, forward and backward digit span and Raven-like matrices. Anthropometry and haemoglobin level measurements were administered to children aged 5–15 years. Anthropometry indicators were also be measured for children aged 2–5 years. As each school is assigned a caterer by the GSFP programme, the sample also included 58 caterers who were interviewed using a semi-structured questionnaire.

### Threats to validity

The main potential threats to the internal validity of the study, including contamination, spill-over effects and Hawthorne-like effects were examined for each of the outcome indicators. From Table 3 it seems that most threats could be avoided by:

i. Assigning treatments to districts rather than to communities within districts in order to avoid contamination effects;
ii. Avoid informing teachers and households of the control communities that the programme will be implemented after 3 years in order to avoid expectancy effects;
iii. Adopt strategies in conducting cognitive and achievement tests that prevent teachers and children from over-performing.

**Table 3:** Threats to internal validity (source: adapted from [12])

Indicator	Metric	Spill-over and contamination	Hawthorne and placebo effects
Schooling	Enrolment, attendance, drop-out and completion	Children may attend school from neighbouring communities to have access to meals	Expectation of coming programme in control communities
Cognitive ability	Raven’s matrices, digit span and/or other tests	Very unlikely	Teachers’ and children’s attempt to over-perform in both project and control communities
Attention	Digit span and/or other test	Very unlikely	Teachers’ and children’s attempt to over-perform in both project and control communities
Learning achievement	Scores on language and maths tests	Very unlikely	Teachers’ and children’s attempt to over-perform in both project and control communities
Physical growth	Anthropometric measures of height and weight	Children from other communities may access school meals	Very unlikely
Physical activity levels (PAL)	Parents’ perceptions	Very unlikely	Very unlikely
Diet diversity	Household consumption	Very unlikely	Very unlikely
Micronutrient intake	Iron status, anaemia	Children from other communities may access school meals	Very unlikely
Income	Farm profits	Unlikely, if food purchases are made in control communities	Very unlikely

Given the panel structure of the data there is a potential risk of differential attrition. However, it is difficult to predict why households or farmers from the control groups should respond to the interviews in different ways. Refusal to take part in the interview by households not benefiting from the project seems to be the main threat. However, as shown in Table 3, the project has limited impact on households’ expectations in both project and control groups and, therefore, should have limited impact on response rates.

### Study area and site selection

Selection of the target areas involved three key steps: 1) the first step involved selecting 58 districts at random within Ghana from a sample frame including all districts in the country. The sample frame was stratified by region, and district inclusion was prioritised using data from the GSFP retargeting exercise including data on the prevalence of poverty and food insecurity; 2) the second step involved identifying 2 comparable schools within each of the 58 selected districts. A list was obtained through the GSFP secretariat including schools not currently covered by the GSFP in each district. Data from the annual school census from 2011 to 2012 were then used to match schools not receiving the GSFP and identify ‘best matched’ pair. The allocation of school feeding and control was then randomised (lottery style) within each pair; 3) the third step in the site selection protocol involved the random allocation of districts to the HGSF+/GSFP groups by modelling pilot selection using a set of community- and district-level variables and selecting the permutation of allocation that minimises the *R*^*2*^ for the predicted selection [13].

### Survey instruments

The impact evaluation includes child-, household-, school-, caterer- and community-level data collection as shown in Table 4.

**Table 4:** Survey instruments

Instrument	Topic/Modules
Household questionnaire	• Household roster (main demographic characteristics, including of children residing elsewhere) • Education (school enrolment, attendance, education of all household members, time spent in class and working, distance and transport to school, meals while in school, parents’ aspirations, PTA membership and involvement) • Household assets and farm assets (household facilities and durables including land and livestock holdings) • Economic activities (simple income questionnaire on time spent working by household members in wage work, own business and own farm) • Expenditure (monetary expenditure and own production of food, education, health, durables, and non-food expenditure) • Anthropometry (height and weight of parents and children above 6 months of age - parents measurements are taken to assess the genetic potential) • Micronutrient status (haemoglobin levels, anaemia prevalence) • Cognitive and literacy and maths achievement tests (test scores on maths, literacy, Raven’s matrices and digit span test) • Farm income (agricultural production and revenues, input expenditure and depreciation of farm assets) • Other income (a simplified income questionnaire for other income sources like microenterprises, transfers, remittances, gifts, etc.)
School questionnaire	• School facilities (school characteristics including boards, toilets, furniture, books and all school-feeding related characteristics - kitchen, storage room, etc.) • School participation (school-level data on enrolment, attendance and drop-out) • School management and food procurement • Teachers (qualifications, living conditions and aspirations) • Training and monitoring activities

*PTA* Parent-teacher Association

### Methods of analysis

The randomised design allows for the identification of causal impacts of interventions using comparisons of mean outcomes between the randomised treatment arms at endline. The analysis will follow the intention-to-treat approach as protocol and as treated, using econometric analysis for all the relevant outcomes of the intervention. Following Bruhn and McKenzie, impact will be assessed for the different treatment arms using both a ‘difference-in-difference’ (DID) estimator and a single difference analysis of covariance (ANCOVA) model [14].

The DID estimate is calculated as the average change in the outcome of interest (*Y*) in the treatment arm (*T*) minus the change in outcome in the control group (*C*), or:

$$ {\Delta}^{DID}=E\left[\left({\overline{Y}}_1^T-{\overline{Y}}_0^T\right)-\left({\overline{Y}}_1^C-{\overline{Y}}_0^C\right)\right]. $$

A difficulty of DID analysis is serial correlation [15] resulting from unobserved factors affecting the outcomes that are themselves correlated over time and that produce auto-correlated errors and invalid standard errors. Serial correlation affects estimated standard errors and can lead to erroneous acceptance or rejection of null hypotheses but not the estimation of the effect size of the intervention. Thus, it may lead to erroneously finding or not finding a statistically significant impact of the intervention. Angrist and Pischke illustrate how this problem can be addressed by calculating clustered standard errors [16], a procedure that is easily implemented using Stata software. Clustered standard errors will also be employed in all cases in which correlated outcomes are observed within the same unit of analysis. For example, when the impact of the intervention is analysed at the school level and test scores within school are obviously correlated. Similarly, clustered standard error will be used at the household level when the project is affecting more than one child within the same family, as in the case of impact on younger siblings.

The single difference model specification has the following form:

$$ {Y}_{i1}={\beta}_0+{\beta}_1{T}_i+{\beta}_2{Y}_{i0}+{\varepsilon}_i, $$

where *Y*_*i*0_ is the outcome variable at baseline, *Y*_*i*1_ is the outcome variable at endline and *T*_*i*_ is a dummy variable for the treatment. The ANCOVA estimator has been shown to provide a more efficient estimate of programme impact when auto-correlation of outcomes is low [14].

As additional robustness checks, depending on the level of clustering of the outcome under analysis, we will employ multi-level regression models that account for the hierarchical nature of the data [17]. Multi-level models, also known as mixed-effects models, use both fixed effects (covariates) and random effects at school and household level.

### Markets

Early studies of food prices in Ghana found negligible price differences across the country [18]. Regional equality of consumer prices, however, does not imply the equality of producer prices at a more localised level. The ability of market interventions to influence local price dynamics depends on the level of spatial market integration between local markets. Abdulai [19] analysed the maize market in Ghana and found a high level of integration, meaning a quick transmission of prices from one locality to the other. In these circumstances large purchases of staple food in localised markets are unlikely to produce price changes. Cudjoe et al. tested for market integration for several staple foods in Ghana and found a high level of integration for rice and maize but much less for tubers such as cassava and yam [20]. Prices of the latter items may be strongly localised and transmission between markets may not be easy. It should also be noted that the studies quoted above looked at market integration across large wholesale markets that are well-connected by roads and communication flows. Differences in prices might emerge in more remote and isolated areas even for more commercial crops like maize and rice. We therefore considered studying the impact of the intervention on local market prices, particularly when the food purchased consists of food items that are not highly commercialised such as cassava and yam.

Impact on prices could, in principle, be observed through the household-level questionnaires. The farm gate price could be observed at the household level by including in the questionnaire questions related to prices paid and time of sales. This, however, would complicate the income section of the farmer questionnaire. Consumer prices are more difficult to observe in a standard household survey because the recall time is 7 or 30 days and there is only one survey per year. As part of the programme monitoring activities, price data will be collected, on a monthly basis, for main staple crops in the local market next to each of the selected schools for a sub-sample of farmer households. Collection of prices does not even require visits to markets if stable contacts can be established with collectors in each of the markets and prices could be communicated by phone.

### Heterogeneity of impact

The large dataset will allow for extensive sub-group analysis, including gender, age and geographic characteristics. The impacts of school feeding in different contexts are quite heterogeneous and context-specific [12]. School feeding, for instance, has been associated with marked improvements school participation by girls in rural areas with large gender disparities in access to education [21]. Smallholder farmers targeted by the programme will, in large proportions, be women. From the educational perspective, school feeding impact has also been found to vary with pupil age, as household schooling decisions are also affected by the opportunity costs of education, that tend to increase with age and vary by gender.

The programme is targeted to disadvantaged groups. The main beneficiaries are located in poor, rural districts of the country and the programme has a potential poverty inequality reduction impact at the national level. At the local level, the programme has a potential poverty reduction impact, but the inequality reduction impact will depend on whether:

- The project will increase enrolment. Children going to school are likely to be from a richer background and from more accessible areas
- The project will involve small farmers. The programme might rely on large farmers or traders for the provision of food

### Cost-effectiveness

Cost data will be collected retrospectively following an ingredients approach using a semi-structured questionnaire. The survey will be based on a standardised costing framework capturing capital (fixed) and recurrent costs incurred at the school level. The questionnaire will also cover both cash and in-kind contributions and will be used to estimate both financial and economic costs. Financial costs capture actual expenditures in terms of programme implementation on an annual basis. Economic costs included the opportunity costs of community members, teaching staff and other school-level stakeholders involved in the school feeding and school health and nutrition (SHN) service provision. Opportunity costs of school staff and community members will be calculated using local pay scales. Capital costs will be annuitised over the useful life of all relevant school-level assets using a discount rate of 3 % as per World Bank recommendations. Annuitisation enables an equivalent annual cost to be estimated and reflects the value in-use of capital items, rather than reflecting when the item was purchased [22].

Process and output data covering the adequacy of the service delivery will be collected from monitoring visits on a quarterly basis using standardised data collection forms. Output data will be combined with the costs to provide estimates of cost-efficiency metrics, including costs per beneficiary, kilocalories, iron, and vitamin A delivered. Sensitivity analysis will be undertaken to account for uncertainties in the economic evaluation. The figures obtained in this way will then be compared to figures calculated for other interventions.

Of particular interest is the cost-effectiveness of the community-level component of the intervention. The comparison between the HGSF+ and the regular GSFP groups is roughly equivalent to the comparison between a ‘home-grown’ school feeding project and a standard school feeding project. Many would expect HGSF to be cheaper and more cost-effective because of lower transport costs. However, the alternative procurement source, its distance and affordability is unknown, and hence the difference in costs between the two programmes is an empirical question.

### Data collection

The enumerators were recruited from Noguchi Memorial Institute for Medical Research (NMIMR) and Institute of Statistical, Social and Economic Research (ISSER), and trained for the baseline survey. Each team, led by a supervisor and assisted by community leaders conducted household listings and sampling in each enumeration area (EA). Maps were obtained for most of the EAs from the Ghana Statistical Service. The EA maps made it possible to identify all dwelling structures within a geographical space with a well-defined boundary. All dwelling/housing structures within each EA were serially numbered to facilitate the complete listing of households. The list of households in each EA constituted the sampling frame from which participating households were selected at random for the interview. A total of 2626 households in 116 communities were surveyed (see Table 5 for the data collection coverage) between the 22 June and the 2 September 2013.

**Table 5:** Household data collection coverage

Region	Communities	Number of households		
		Intervention	Control	Total
Western	8	96	80	176
Central	6	75	60	135
Greater Accra	2	24	25	49
Volta	10	123	101	224
Eastern	6	75	60	135
Ashanti	18	225	180	405
Brong-Ahafo	12	150	120	270
Northern	26	319	284	603
Upper East	10	225	179	404
Upper West	10	125	100	225
Total	116	1437	1189	2626

In each household, all children aged between 5 and 15 years were asked education-related questions (enrolment, attendance, drop-out) and were tested in literacy, maths, forward and backward digit span and Raven’s matrices. Anthropometry measurements were undertaken for children aged 2–15 years. Tests and measurements were made at the household level because not all the children in the targeted schools resided in the selected localities where the schools were situated. Height measurements were taken with Leicester Height Measures and weights were measured using Tanita Electronic Scales WB-100A/WB-110A Remote Display Version scales, which allow height measurements of up to 2 m 10 cm to the nearest 1 mm. The height and weight measures were assembled and placed on a level surface. In the absence of a level ground in the household, a suitable place was identified for the measurement in the community. A sub-set of children aged 5–15 years were randomly selected for haemoglobin and parasitology measurements. Haemoglobin levels were collected using HemoCue Hb 201+ analyser, with standard controls reagents (Hemotrols) used to verify appropriate device function on a daily basis.

### Data management and analysis

All questionnaires were checked in the field for consistency and completeness by field supervisors before data entry. Data were entered in CSPro and later transferred to Stata 12 for data cleaning and analysis. Simple frequency tables of variables from each module in the questionnaire were generated from the database and examined for inconsistencies. Errors related to wrong entries were verified from the specific questionnaire and corrected appropriately.

### Ethical approval

Ethical clearance was obtained from the Institutional Review Board of the Noguchi Memorial Medical Research Institute of the University of Ghana and sought at the Imperial College Research Ethics Committee. Meetings were held from early stages in the study development with relevant government ministries both at central and decentralised levels to discuss the purpose, procedures and risks involved in the study. Informed consent was obtained from parents/guardians of children through written and verbal information provided before interviews.

## Results

Table 6 summarises the characteristics for key variables of interest in the study population and by study group. We also report the main evaluation comparisons, including school feeding (combined GSFP and HGSF) versus control (no school feeding), regular school feeding (GSFP) versus HGSF (combined HGSF+ and HGSF++) and HGSF with micronutrient sprinkles (HGSF++) versus HGSF without sprinkles (HGSF+). The tests of balance show evidence of small differences across the treatment arms for several variables across education, nutrition, agriculture and other socio-economic domains. In addition, approximately 19 % of children in the target age group (5–15 years) received some form of free school meals at baseline. Of the total 8407 children aged 15 years or younger, 48 % were girls.

**Table 6:** Key baseline characteristics of participants for all individuals and households by study group, Ghana baseline survey

			Control (C)		School feeding (SF)								
					Ghana SF Programme (GSFP)		‘Home-grown’ school feeding (HGSF)				Main evaluation comparisons*		
	All						HGSF+		HGSF++		Ha: [*A − B*] ! = 0, Pr(|*T*| > |*t*|)		
Characteristic	*n*	Estimate	*n*	Estimate	*n*	Estimate	*n*	Estimate	*n*	Estimate	[SF − C]	[GSFP − HGSF]	[HGSF+ − HGSF++]
Outcomes													
Absentee days over last 7 days	6217	0.130 [0.678]	2754	0.121 [0.672]	1765	0.117 [0.613]	855	0.186 [0.790]	843	0.130 [0.685]	0.327	0.0745	0.1227
Age started school (aged 5–15 years)	3907	7.18 [1.95]	1734	7.16 [1.92]	1105	7.36 [1.88]	560	7.18 [2.05]	508	6.86 [2.07]	0.5276	0.0001	0.0123
Number of times repeated a class	6067	0.23 [0.62]	2677	0.21 [0.57]	1726	0.25 [0.62]	825	0.26 [0.67]	839	0.22 [0.72]	0.0356	0.7323	0.2238
Maths test score	5826	2.51 [2.79]	2588	2.31 [2.70]	1646	2.54 [2.75]	801	2.91 [3.04]	791	2.74 [2.87]	<0.001	0.004	0.2756
Literacy test score	5849	3.06 [3.69]	2596	2.75 [3.50]	1661	3.05 [3.56]	804	3.58 [4.07]	788	3.57 [4.06]	<0.001	<0.001	0.9423
Raven’s test score	5830	4.46 [2.74]	2590	4.32 [2.75]	1650	4.52 [2.69]	800	4.84 [2.70]	790	4.41 [2.81]	<0.001	0.2448	0.002
Digit span score	5883	4.78 [2.40]	2615	4.56 [2.40]	1664	4.89 [2.37]	809	5.13 [2.39]	795	4.89 [2.39]	<0.001	0.152	0.0487
Height-for-age (5–15 years) z-score	5232	−0.925 [1.35]	2303	−0.943 [1.43]	1494	−0.963 [1.29]	730	−0.888 [1.24]	705	−0.827 [1.31]	0.3999	0.0268	0.3666
BMI-for-age (5–15 years) z-score	5232	−0.592 [0.924]	2303	−0.575 [0.964]	1494	0.636 [0.895]	730	−0.574 [0.857]	705	−0.570 [0.912]	0.2542	0.051	0.9284
Haemoglobin levels (g/dL)	714	11.3 [1.34]	422	11.3 [1.35]	169	11.3 [1.36]	32	11.4 [1.47]	91	11.3 [1.21]	0.9088	0.8764	0.8469
Total maize production volumes (kg)	2626	787 [1751]	1163	864 [2034]	722	807 [1596]	375	590 [1248]	366	702 [1478]	0.0439	0.0376	0.2646
Total rice production volumes (kg)	2626	141 [652]	1163	149 [700]	722	148 [645]	375	137 [678]	366	111 [449]	0.6003	0.4551	0.5297
Total maize sale volumes (kg)	2626	393 [1196]	1163	432 [1261]	722	446 [1337]	375	270 [880]	366	292 [907]	0.1381	0.0057	0.7435
Total rice sale volumes (kg)	2626	84 [484]	1163	94 [583]	722	87 [413]	375	70 [397]	366	61 [327]	0.3509	0.2895	0.7286
Other variables													
Age (for 15 years and younger)	8407	7.5 [4.2]	3153	5.8 [9.0]	1918	6.2 [9.5]	942	6.2 [9.3]	917	6.1 [9.9]	0.057	0.847	0.8071
Is a girl? (for 15 years and younger)	8407	0.48 [0.5]	3799	0.48 [0.50]	2318	0.49 [0.50]	1167	0.47 [0.50]	1123	0.51 [0.5]	0.4933	0.9377	0.0989
Birth order (for all children)	8533	2.9 [2.0]	3791	2.9 [2.1]	2397	3.0 [2.0]	1189	3.0 [2.0]	1156	2.9 [1.9]	0.8275	0.021	0.2204
Is enrolled in school? (for 5–15 years)	6178	0.92 [0.27]	2755	0.91 [0.29]	1737	0.92 [0.26]	852	0.94 [0.01]	834	0.91 [0.29]	0.0375	0.9395	0.0065
Receives free school meals?	6280	0.19 [0.39]	2809	0.17 [0.38]	1768	0.23 [0.42]	860	0.13 [0.34]	843	0.24 [0.43]	0.0001	0.0055	<0.001
Distance to nearest school	6343	0.47 [3.24]	2765	0.45 [3.14]	1808	0.58 [4.34]	891	0.21 [0.85]	879	0.59 [2.29]	0.597	0.0992	<0.001
Time to school	6333	21.4 [45.6]	2778	19.6 [31.3]	1796	18.8 [32.5]	888	17.6 [22.7]	871	35.9 [95.1]	0.007	<0.001	<0.001
Total education expenditure	5635	23923 [35642]	2410	19250 [30878]	1640	27089 [37899]	789	25732 [37096]	796	29752 [40791]	<0.001	0.6249	0.0403
Mother’s education level (5–15 years)	5096	5.9 [9.4]	2290	5.7 [9.1]	1437	6.1 [9.6]	690	6.1 [9.4]	679	6.0 [9.8]	0.1946	0.8518	0.7608
Education level of head of household	2626	1.4 [2.6]	1163	1.4 [2.7]	1463	1.3 [2.5]	375	1.6 [2.7]	366	1.3 [2.2]	0.9393	0.2147	0.1344
Household size	2626	5.7 [2.4]	1163	5.7 [2.4]	722	5.8 [2.4]	375	5.7 [2.3]	366	5.6 [2.3]	0.8747	0.1774	0.5572
Per-capita expenditure quintile	2625	3.1 [1.4]	1162	3.2 [1.4]	722	2.9 [1.4]	375	3.3 [1.4]	366	3.1 [1.4]	0.1763	0.0008	0.2199
Household expenditure on food	2626	3473 [2187]	1163	3483 [2213]	722	3370 [2254]	375	3502 [1846]	366	3608 [2289]	0.815	0.1041	0.4874
Household expenditure on health	2626	93.5 [112]	1163	97.7 [116]	722	88.7 [107]	375	93.3 [108]	366	90.0 [112]	0.0894	0.595	0.6768
Household expenditure on education	2626	214 [333]	1163	204 [328]	722	195 [311]	375	233 [340]	366	260 [374]	0.1926	0.0034	0.3013
Household expenditure on transport	2626	317 [415]	1163	315 [407]	722	297 [393]	375	352 [447]	366	326 [447]	0.8157	0.0607	0.4316
% share of HH expenditure spent on food	2626	59.8 [15.8]	1163	59.6 [16.4]	722	60.3 [15.4]	375	58.0 [14.0]	366	61.1 [16.0]	0.5613	0.3073	0.0049

Estimates are expressed as mean and standard deviation

*BMI* body mass index, *HH* household. * T-test statistics for comparisons across treatment arms

In the education domain, 92 % of children aged 5–15 years were enrolled in school, and mean enrolment rates were marginally lower in the control population (0.91, SD 0.29) compared to the school feeding group (0.93, SD 0.26). Significant differences were also found for age of first enrolment, the number of times that a year was repeated, and across all the four test scores.

In the nutrition domain for children aged 5–15 years, the mean z-scores for the anthropometrics measures of height for age and BMI for age were −0.925 (SD 1.35) and −0.592 (SD 0.924) respectively, with significant differences across the GSFP versus HGSF comparison groups. Iron status, as measured through haemoglobin levels, for the sub-sample of children (*n* = 714) who were assessed, was on average 11.3 g/dL (SD 1.34), just below the 11.5 g/dL cut-off for non-anaemia in the 5–11 years age group.

In terms of household socio-economic characteristics, there were neither significant differences among the treatment groups for the mean education levels of mothers and household heads, nor for household size. There was, however, a significant difference in terms of per-capita household expenditure quintiles between households in the GSFP and HGSF groups, but no other substantive differences with regards to household expenditure were observed.

In the agriculture domain, across the survey population the mean production of maize over the previous 12 months was 787 kg (SD 1751), with average household sales of maize during the same period of 393 kg (SD 1196). Mean household production of rice was 141 kg (SD 625), with average annual sale volumes of 84 kg (SD 484). Significant differences were found across treatment arms in terms of maize production and sales.

## Discussion

School feeding interventions are implemented in nearly every country in the world, with the potential to support the education, health and nutrition of school children from low-income households [23]. To date, there is little evidence on the potential for agriculture and community development. This paper described the design and baseline results for a randomised evaluation of school meals interventions linked to smallholder agriculture. As far as we are aware, it is the first to examine explicitly from a holistic perspective the simultaneous impact of a national school meals programme on micronutrient status, alongside outcomes in nutrition, education and agriculture domains. The evaluation builds on a trial design taking place in Mali that includes an extensive analysis of the programme theory for the intervention. As the intervention is complex, the scope of this evaluation is also very broad and includes measurement of a range of outcome indicators across multiple traditional disciplines. Designing and implementing such an evaluation is in itself a complex undertaking, involving a multi-disciplinary research team working in close collaboration with programme- and policy-level stakeholders. The survey also required a range of different expertise in the enumeration teams in order to collect data including anthropometry, haemoglobin levels, and educational tests, alongside expenditure, income and other socio-economic-related modules. The use of the survey tools required to capture the data was inevitably fairly time-intensive. Extensive analysis of the rich baseline data is currently underway.

A number of important considerations can be drawn from the baseline data analysis. Firstly, the tests of balance showed evidence of small differences across the treatment arms for several variables across education, nutrition, agriculture and other socio-economic domains. The randomisation of treatment across the arms of the cluster-randomised trial is aimed at minimising the systematic differences in the outcomes between the intervention groups. In practice, differences between the intervention groups can arise due to sampling error in moderate sample sizes. When estimating programme impact it is important to control for these differences where they exist.

In addition, approximately 19 % of children in the target age group (5–15 years) received some form of free school meals at baseline. Similar findings were reported in a similar study in Mali in 2013 by Masset and Gelli [13] where, because of information flow constraints, the original list of schools used in the randomisation included schools with school feeding. This finding has important implications in terms of the evaluation design, considerably reducing sample sizes available for comparisons after the follow-up survey. The small sample sizes between the HGSF+ and HGSF++ comparisons are a particular concern, and power calculations using the baseline data suggest folding these two arms into one, adding micronutrient sprinkles to the HGSF+ intervention.

Significant differences were found in the means of a number of outcome and control variables across the intervention groups. It appears, therefore, that at baseline the random allocation process did not achieve statistically comparable treatment groups. In particular, important differences across groups include marketed surplus, livestock income, per capita food consumption and intake, school attendance, anthropometric status in the 2–5 and 5–15 years age groups. Differences in outcome and control variables across groups will be controlled when estimating treatment effects. More in-depth analyses of the very rich baseline dataset, examining the associations between key outcomes and variables along the complex agriculture-nutrition are also underway.

## Conclusions

Assessing the simultaneous impact of ‘home-grown’ school feeding on micronutrient status, health, education and agriculture is a complex undertaking, involving coordination across policy, programme and research stakeholders. This study is the first to examine the effects of alternative implementation modalities of school meals on nutrition, health education and agriculture in Ghana. The findings of this evaluation will provide important evidence to support policymakers in the scale-up of the national programme.

## Figures and Tables

**Fig. 4:**
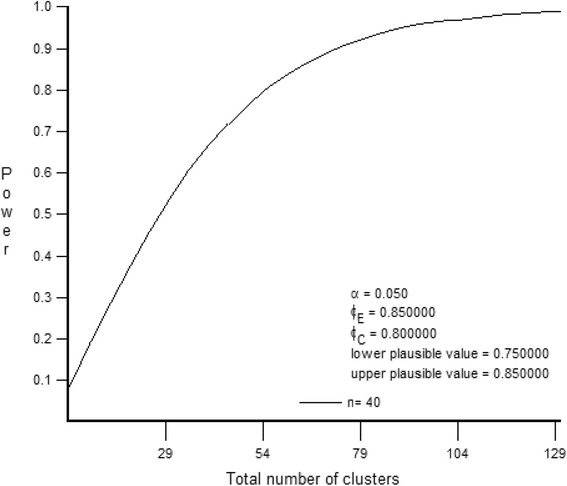
School attendance: Power versus number of clusters

**Fig. 5:**
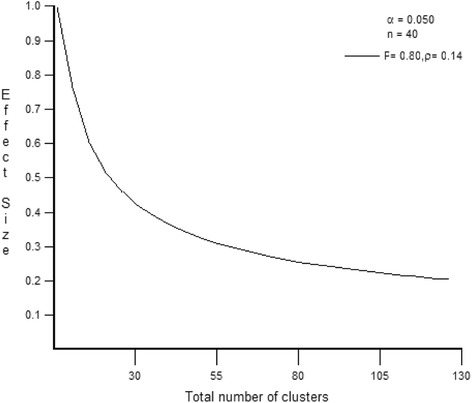
Raven’s tests: minimum detectable difference versus number of clusters

**Fig. 6:**
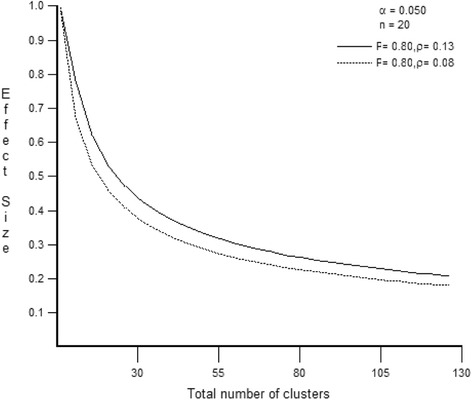
Haemoglobin: minimum detectable difference versus number of clusters

**Fig. 7:**
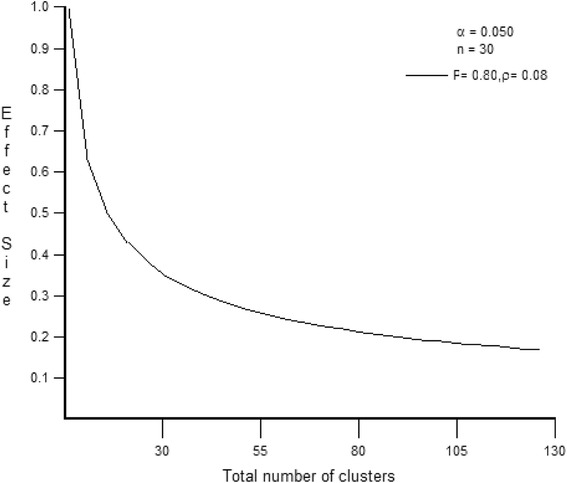
Height-for-age *z*-scores (HAZ): minimum detectable difference versus number of clusters

**Fig. 8:**
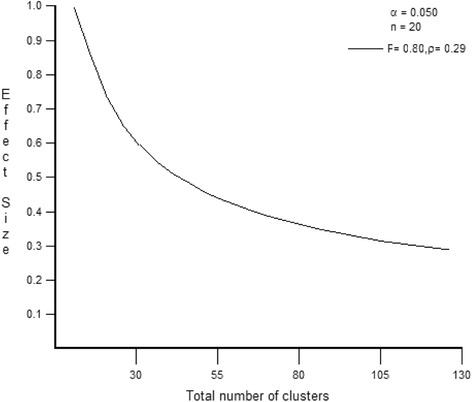
Farm incomes: minimum detectable difference versus number of clusters

## Appendix 1

### Power calculations

#### School attendance

We used the rural sample of the GDHS data of 2008 to estimate attendance rates of children in the age group 6 to 14 and we found rates of 79 % for boys and 81 % for girls. The chart below plots values of power for increasing number of clusters assuming a project impact of 5 percentage points on attendance rates in primary school. A sample of just 60 clusters and collecting data on 40 children is sufficient to detect such an impact with 80 % statistical power (Fig. 4).

#### Cognitive tests

We obtained data on outcomes of cognitive tests from a sample of rural children tested in 2003 using Raven’s matrices. The average score on the test was 15.3 out of 36 questions with a SD of 5.9 and an intracluster correlation coefficient (ICC) of 0.14. The chart below plots that minimum detectable difference against the number of clusters. We assumed a number of 40 children per cluster considering 20 households interviewed in each cluster and an average of 2.3 children in the relevant age group per each household with children (Fig. 5).

The table below summarises the standardised detectable differences and corresponding absolute values of the tests for different study designs (Table 7).

**Table 7:** Raven’s tests: standardised detectable differences and equivalent levels for different designs

	Children aged 6 to 14 (in SDs)	Equivalent level
30 clusters	0.43	2.5
60 clusters	0.30	1.8
120 clusters	0.21	1.2

*SD* standard deviation

#### Anaemia

Data for power calculations were obtained from the 2008 GDHS. We calculated means, SDs, and ICCs for rural children aged 6 months to 5 years and rural mothers aged 15–49 years. See the tables below for the level of haemoglobin and prevalence rates of any anaemia (including severe, moderate and mild) (Table 8).

**Table 8:** Haemoglobin levels and anemia prevalence rates (rural mothers and under-5 s)

	Mean	SD	ICC	Observations.
Children (haemo)	9.22	1.73	0.126	1473
Children (prevalence)	0.854	0.35	0.045	1473
Mothers (haemo)	11.9	1.78	0.080	2681
Mothers (prevalence)	0.619	0.49	0.052	2681

*ICC* intracluster correlation coefficient, *SD* standard deviation

The chart below plots the minimum detectable difference in terms of SDs from the mean for children (ICC = 0.13) and mothers (ICC = 0.08). In both cases it is assumed that the size of the sample in each cluster is 20. This is consistent with 20 household interviews per community and considering that several children may end up not being tested. In any case, only a marginal gain can be obtained by expanding the sample beyond 20 as power is mainly driven by the number of clusters (Fig. 6).

The table below reports the standardised detectable differences and their equivalent level values for 3 different designs: 30 clusters, 60 clusters and 120 clusters. In each case 50 % of the sample is allocated to the project group. Differences between groups of mothers can be estimated more precisely because the ICC is lower for mothers though the sample variance is slightly larger (Table 9).

**Table 9:** Haemoglobin: standardised detectable differences and equivalent levels for different designs

	Children (in SDs)	Equivalent level	Mothers (in SDs)	Equivalent level
30 clusters	0.44	0.76	0.38	0.65
60 clusters	0.31	0.56	0.26	0.46
120 clusters	0.21	0.36	0.18	0.32

*SD* standard deviation

#### Child nutrition

We used data from the GDHS 2008 to estimate mean and SD of height-for-age *z*-scores of rural children and we found these to be −1.03 and 1.57 respectively. The ICC is 0.08. The chart below plots the standardised minimum detectable difference against the number of clusters assuming a sample of 30 children measured in each community (Fig. 7).

The table below summarises the values of the standardised and equivalent absolute values of the detectable differences (Table 10).

**Table 10:** Height-for-age *z*-scores (HAZ): standardised detectable differences and equivalent levels for different designs

	Children under 5 years (in SDs)	Equivalent level
30 clusters	0.35	0.55
60 clusters	0.25	0.39
120 clusters	0.17	0.27

*SD* standard deviation

#### Farm income

We used data from GLSS4 of 1998/1999 to estimate average farm income of rural households (1200 cedis) and relative SD (1400 cedis). We found an extremely high ICC. Income is the most difficult outcome to estimate with sufficient precision. The chart below plots the standardised minimum detectable difference against the number of clusters assuming 20 farmers interviewed in each community. Since the SD is roughly similar to the mean the vertical axis can be interpreted as a percentage difference (Fig. 8).

The table below summarises the standardised differences and the corresponding percentage changes in income that can be estimated with different study designs (Table 11).

**Table 11:** Farm income: standardised detectable differences and equivalent levels for different designs

	Farm incomes (in SDs)	Percentage difference
30 clusters	0.6	0.69
60 clusters	0.4	0.46
120 clusters	0.3	0.35

*SD* standard deviation

## Abbreviations

CGIAR: Consultative Group of International Agricultural Research
DICs: District Implementation Committees
EA: enumeration area
GDHS: Ghana Demographic and Health Surveys
GDP: gross domestic product
GSFP: Ghana School Feeding Programme
Hb: haemoglobin
HGSF: ‘home-grown’ school feeding
HGSF+: ‘home-grown’ school feeding pilot
HGSF++: ‘home-grown’ school feeding pilot plus micronutrient sprinkles
MoLGRD: Ministry of Local Government and Rural Development
PAL: physical activity levels
SD: standard deviation
SIC: School Implementation Committee
WFP: World Food Programme
WHO: World Health Organisation
